# Multimorbidity due to novel pathogenic variants in the *WFS1/RP1/NOD2* genes: autosomal dominant congenital lamellar cataract, retinitis pigmentosa and Crohn’s disease in a British family

**DOI:** 10.1136/bmjophth-2023-001252

**Published:** 2023-07-14

**Authors:** Vanita Berry, Alexander Ionides, Michalis Georgiou, Roy A Quinlan, Michel Michaelides

**Affiliations:** 1 Genetics, UCL Institute of Ophthalmology, University College London, 11-43 Bath Street, London, EC1V 9EL, UK, London, UK; 2 Moorfields Eye Hospital NHS Foundation Trust, London, EC1V 2PD, UK, London, UK; 3 Department of Biosciences, University of Durham, Upper Mountjoy Science Site, Durham DH1 3LE, UK, Durham, UK

**Keywords:** Vision, Genetics, Lens and zonules, Retina

## Abstract

**Background:**

A five generation family has been analysed by whole exome sequencing (WES) for genetic associations with the multimorbidities of congenital cataract (CC), retinitis pigmentosa (RP) and Crohn’s disease (CD).

**Methods:**

WES was performed for unaffected and affected individuals within the family pedigree followed by bioinformatic analyses of these data to identify disease-causing variants with damaging pathogenicity scores.

**Results:**

A novel pathogenic missense variant in *WFS1*: c.1897G>C; p.V633L, a novel pathogenic nonsense variant in *RP1*: c.6344T>G; p.L2115* and a predicted pathogenic missense variant in *NOD*2: c.2104C>T; p.R702W are reported. The three variants cosegregated with the phenotypic combinations of autosomal dominant CC, RP and CD within individual family members.

**Conclusions:**

Here, we report multimorbidity in a family pedigree listed on a CC register, which broadens the spectrum of potential cataract associated genes to include both *RP1* and *NOD2*.

**What is already known on this topic**

- Cataract is a clinically and genetically heterogeneous disease, displaying a broad range of clinical phenotypes, but thus far characterisation of any genetic association between cataract and other multimorbidities has not been reported.

**What this study adds**

- There is a genetic basis to the multimorbidities of retinitis pigmentosa (RP) and Crohn’s disease that accompany congenital cataract in the five-generation family under investigation. *WFS1, NOD2* and *RP1* variants combine to associate appropriately with the three observed multimorbidities within the family.

**How this study might affect research, practice or policy**

- The discovery of the genetic basis for the three observed multimorbidities by variants of the three different genes suggests detailed biochemical and cell biological studies are required to determine the nature of the mechanistic link(s) between *WFS1*, *NOD2* and *RP1*. Calcium homeostasis and the stress response are potential targets for such initial studies.

## Introduction

**Figure 1:**
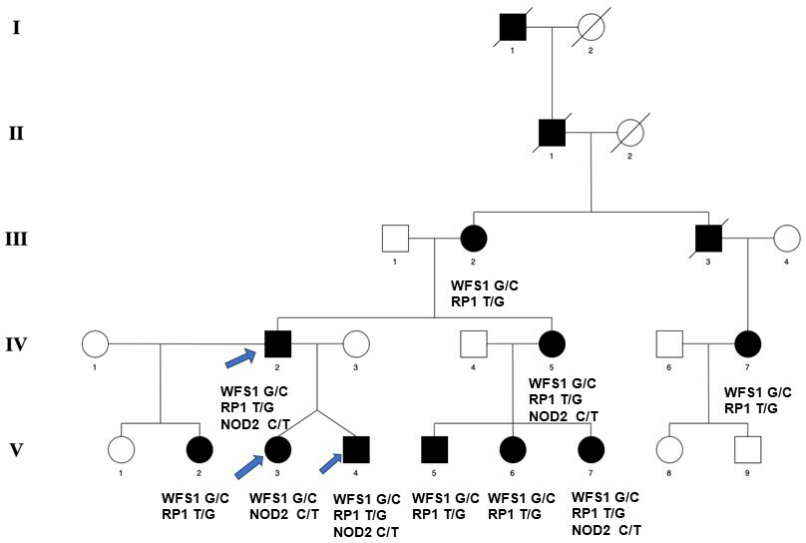
Abridged pedigree presenting with lamellar CC, RP and CD. Squares and circles symbolise male and female family members, respectively. Open and filled symbols indicate unaffected and affected individuals. All affected individuals had cataract; individuals III-2, IV-2, IV-5, IV-7, V-2, V-4, V-5, V-6 and V-7 had RP and individuals IV-2, IV-5, V-3, V-4 and V-7 had *NOD2* variant for CD. Arrows indicate those family members who participated in the WES analysis. CC, congenital cataract; CD, Crohn’s disease; NOD, nucleotide oligomerization domain; RP, retinitis pigmentosa; WES, whole exome sequencing.

Next generation sequencing (NGS) has allowed the identification of digenic or trigenic inheritance. We have encountered a rare instance of coexistent autosomal dominant congenital cataract (CC), retinitis pigmentosa (RP) and Crohn’s disease (CD) in affected members of a pedigree that was initially investigated for CC that presented in a 4 year old. Whole exome sequencing (WES) identified a *WFS1* variant causing CC in all the affected individuals, a *RP1* variant causing RP in all but one affected family member and a *NOD2* variant found in five affected family members with CD, thus far confirmed clinically in one member of the pedigree (figure 1). CC was the first autosomal disease to be genetically mapped in humans and it is detected either at birth or during the first decade of life.^1 2^ CCs are mostly inherited as autosomal dominant, but recessive and X-linked inheritance also occur. Nearly 50 cataract-causing genes have been identified (https://cat-map.wustl.edu/).^1 2^ Cataract is, therefore, a clinically and genetically heterogeneous disease, displaying a range of phenotypes.^1 2^ It can be both isolated and associated with other ocular defects such as anterior segment mesenchymal dysgenesis, glaucoma, microcornea and aniridia, or with syndromic conditions such as congenital adrenal hyperplasia^3^ and Wolfram syndrome (WS),^4^ as well as a consequence of metabolic disease such as diabetes.^2^ Thus far the reported genetic association with CC has involved single genes, but NGS allows more complex inheritance patterns to be identified.

Here, we report the multigenic inheritance of three heterozygous variants *WFS1* p.V633L, *RP1* p.L2115* and *NOD2* p.R702W in a British pedigree with lamellar CC, RP and CD. All three genes play crucial roles in ocular tissues. For instance, pathogenic variants in *WFS1* cause WS, isolated CC and optic atrophy. *RP1* variants cause RP, also a clinically and genetically heterogeneous group of progressive inherited retinal dystrophies in which cataract is also mentioned as a clinical phenotype. Finally, *NOD2*, a member of a large family of nucleotide oligomerization domain (NOD) like receptors (NLRs) that are multidomain cytosolic receptors that form signalling complexes to mediate major cellular pathways such as autophagy, inflammation and the innate immune response to cellular injury and stress.^5^ *NOD2* is expressed in the eye^6^ and heterozygous variants can cause cataract.^7^ The NLR family of receptors are expressed in eye tissues during pathological conditions suggesting a role in dry eye, ocular infection, retinal ischaemia, cataract, glaucoma, age-related macular degeneration, diabetic macular oedema and diabetic retinopathy. Moreover, variants in *NOD2* result in a defective response to microbiota in the intestinal tract, which have been proposed to lead to CD, an inflammatory bowel disease^8^ linking *NOD2* mutations to a diverse set of pathologies involving both the colon and the eye. Our data provide genetic evidence for potential overlap in the consequences of mutations in *WFS1, RP1* and *NOD2* to account for the observed multimorbidities of CC, RP and CD within the family pedigree.

## Materials and methods

### Phenotyping

Patients in this pedigree were identified via the proband attending the Genetic Service at Moorfields Eye Hospital, London, UK. All the family members participating in this study underwent a full ophthalmic examination and affected individuals were diagnosed as having isolated bilateral CC with lamellar phenotype (figure 1). CC was first diagnosed (IV-7) at the age of 4 years old. This individual underwent their first treatment with needling at the age of 16 years to her right eye. At the age of 19 years, she had needling to her left eye. Detailed clinical information of the RP and CD phenotypes was sadly unavailable despite our best efforts. The family presented over 30 years ago with lamellar CC as the presenting clinical feature. Advanced phenotyping of family members in the context of this study was not performed because the members were unavailable for follow-up. Phenotyping was performed via a detailed family medical history taken from individual IV-5. Medical records were unavailable to verify the information relayed by this individual. The RP phenotype is late onset, becoming apparent in affected individuals in their 20s. CD was clinically diagnosed in one individual of the pedigree again in their 20s.

### WES and bioinformatics analysis

Genomic DNA was extracted from EDTA-treated blood samples using the Nucleon II DNA Extraction Kit (Scotlab Bioscience, Strathclyde, Scotland, UK). The DNA samples were sequenced at Macrogen Europe. Exon capture and target enrichment was performed using the SureSelectXT Human All Exon V6 post (Agilent, Santa Rosa, CA, USA). Paired-end sequencing was performed on an Illumina Hiseq 2500 high-throughput sequencer, generating mean exome coverage of 50×. Raw data in the ‘fastq’ format was aligned to the UCSC Genome Browser GRCh37/hg19 human reference sequence and analysed using the Phenopolis bioinformatics platform. We used a tiered approach to prioritise rare coding variants using Kaviar (http://db.systemsbiology.net/kaviar/) and Genome Aggregation Database (GnomAD http://gnomad.broadinstitute.org/) or rare variants (GnomAD allele frequency<0.0001) in all the known cataract genes (https://cat-map.wustl.edu/). The variants were then filtered by Combined Annotation Dependent Depletion (CADD) score, with those CADD scores >15 then predicted to be moderately or highly damaging with the highest at the top for both known and unknown genes for cataracts. The Varsome platform was also used for bioinformatic analyses (https://varsome.com).

### Sanger sequencing

Direct Sanger sequencing was performed to validate variants identified by WES for both affected and unaffected family members. Genomic DNA was amplified by PCR using GoTaq 2X master mix (AB gene; Thermo Scientific, Epsom, UK) and WFS1 specific primers (forward primer: 5′ CATCGGCTACTTCCTCTTCC; reverse primer: 5′ AGCAGCTTAAGGCGACAGAG, *RP1*: forward primer: 5′ ttggggttagaggaagaagg; reverse primer: 5′ cctgagtctgtaattgttggaaaa and *NOD2*: forward primer: 5′ TCTTTGCCGCGTTCTACCT; reverse primer: 5′ GCCAATGTCACCCACAGAGT) were designed with Primer3 (http://bioinfo.ut.ee/primer3-0.4.0/). PCR conditions were as follows: 94°C for 5 min for the initial denaturation followed by 30 amplification cycles comprising 30 s at 94°C (denaturing), 30 s at 60°C (annealing) and finally 45 s at 72°C (extending). After cleaning, the PCR products were reacted with BigDye Terminator v3.1, they were run on ABI 3730 Genetic Analyzer (both from Applied Biosystems, Foster City, CA, USA) and analysed using SeqMan Pro (V.8.0.2 from DNASTAR) sequence analysis. After validation, the segregation of each variant was performed for all available affected and unaffected family members.

## Results

**Figure 2:**
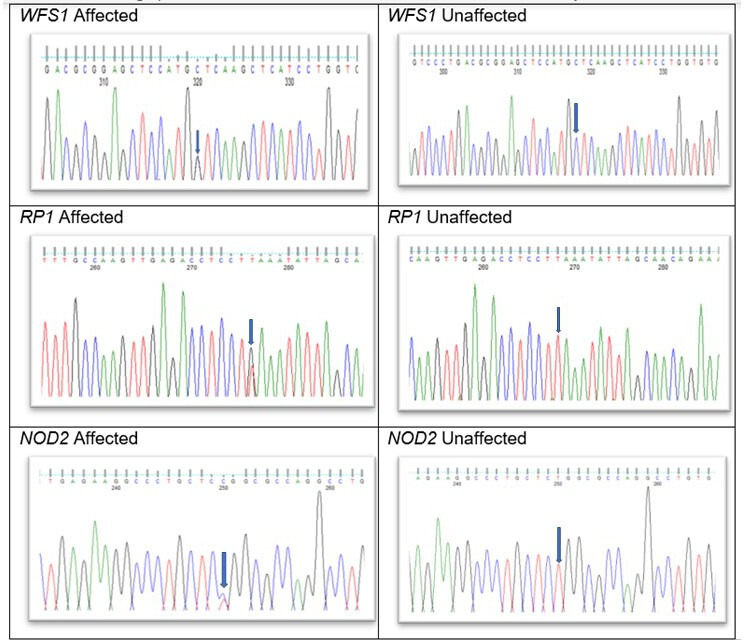
Variant identification by sequence analysis: (a) *WFS1*–missense variant c.1897G>C in affected member, (b) *WFS1* wild type in unaffected members; (c) *RP1*–nonsense variant at c.6344T>G in affected members; (d) wild type *RP1* in unaffected members; (e) *NOD2*–missense variant c. 2104C>T in affected member and (f) *NOD2* wild type in unaffected members. NOD, nucleotide oligomerization domain; RP, retinitis pigmentosa.

A five-generation English pedigree with 10 affected, 3 unaffected and 6 spouses presented with bilateral lamellar CC (figure 1, figure 2A–F, table 1 and table 2). This family was first examined clinically nearly 30 years ago and was found then to present with isolated lamellar CC in all the affected individuals. At that time, it was not possible to map by linkage studies, but now three DNA samples (IV-2, V-3 and V-4) became available for WES. Using the Phenopolis genetic variant analysis pipeline, variants were then filtered by allele frequency. Fifty from a total of 3064 rare coding variants in individual IV-2, 79 rare variants out of 2831 in V-3, and 58 rare variants out of 2925 for individual V-4 remained.

**Table 1:** Pathogenicity scores of variants in *WFS1, RP1* and *NOD2* genes

Genes	Genomic pos./exon	HGVSp	MutationTaster/verdict	REVEL	GERP	CADD	SIFT	DANN
***WFS1***	Chr4p16.1/ Ex-8/8	p.V633L	Disease-causing/0.81-likely pathogenic	Pathogenic/0.90	5.4	26.3	Damaging	0.997
***RP1***	Chr8q12.1/Ex-4/4	p.L2115*	Disease-causing/0.81-pathogenic	Pathogenic/0.96	4.73	35.1	Damaging	0.984
***NOD2***	Chr16q12.1 Ex-4/12	p.R702W	VUS-likely pathogenic	Likely pathogenic/-	–	24.4	Damaging	–

CADD, Combined Annotation Dependent Depletion; DANN, Deep learning approach for Annotation Neural Network; GERP, Genomic Evolutionary Rate Profiling; HGVSp, Human Genome Variation Society (protein); NOD, nucleotide binding oligomerization domain; REVEL, Rare Exome Variant Ensemble Learner; RP, retinitis pigmentosa; SIFT, Sorts Intolerant From Tolerant; VUS, Variant of Uncertain Significance.

**Table 2:** Genotype/phenotype (CC, RP and CD) correlation in affected family members

Affected family members	Genotype: *WFS1*/*RP1*/*NOD2*	Phenotype: CC/RP/CD	Predicted phenotype
III-2	*WFS1*/*RP1*	CC/RP	CC/RP
IV-2	*WFS1*/*RP1*/*NOD2*	CC/RP/-	CC/RP/CD
IV-5	*WFS1*/*RP1*/*NOD2*	CC/RP/-	CC/RP/CD
IV-7	*WFS1*/*RP1*	CC/RP	CC/RP
V-2	*WFS1*/*RP1*	CC/RP	CC/RP
V-3	*WFS1*/*NOD2*	CC/-	CC/CD
V-4	*WFS1*/*RP1*/*NOD2*	CC/RP	CC/RP/CD
V-5	*WFS1*/*RP1*	CC/RP	CC/RP
V-6	*WFS1*/*RP1*	CC/RP	CC/RP
V-7	*WFS1*/*RP1*/*NOD2*	CC/RP/CD	CC/RP/CD

CC, congenital cataract; CD, Crohn’s disease; NOD, nucleotide binding oligomerization domain; RP, retinitis pigmentosa.

The top scoring variant by CADD was a rare heterozygous variant NM_006269.2: c.6344T>G; L2115* in exon 4 of *RP1* with a score of 35.1 and NM_006005.3: c.1897G>C; p.V633L in exon 8 of *WFS1* with a score of 26.3 (figure 2, table 1). Individual (IV-2) harboured *WFS1, RP1* and *NOD2* gene variants, his son (V-4) also inherited all three variants (figure 2, table 1). His daughter (V-3) inherited the *WFS1* and *NOD2* variants. Direct Sanger sequencing confirmed both variants (figure 2) and they cosegregated in other affected family members. Subsequently, we found out that many of the affected members of the family had RP (figure 1, table 2). We reviewed our WES data for the genes associated with CD and found a likely pathogenic missense variant NM_022162.3: c.2104C>T; p.R702W with a CADD score of 24.1 in exon 4 of the *NOD2* gene in individuals IV-2, IV-5, V-3, V-4 and V-7. Individual V-7 developed very severe CD symptoms in her early 20s (figure 2, table 1,table 2). This variant is highly frequent in the Western population and can be associated with CD, although there might also be population-dependent component, for example, Zaahl *et al*.^9^ None of the variants in *WFS1*, *RP1* or *NOD2* were found in unaffected family members.

Rare Exome Variant Ensemble Learner tool was used to predict the pathogenicity of missense variants based on a combination of scores from 13 individual tools; Genomic Evolutionary Rate Profiling Neutral Rate corresponds to the neutral rate conservation score of the site and CADD is a score for the deleteriousness of a variant. A CADD score of >15 is considered damaging; Sorts Intolerant Frome Tolerant score (0.0–0.05) to check the deleteriousness of the amino acid substitution on the protein function; * indicates protein truncation as a result of the sequence change.

## Discussion

Here, we report heterozygous variants in *WFS1, RP1* and *NOD2* associated with the multimorbidities of CC, RP and CD. These multimorbidities presented in a five-generation family pedigree that was initially identified some 30 years ago with autosomal dominant CC.

In WS, compound heterozygosity is often observed particularly within the C-terminal tail domain.^10^ There are three other *WFS1* variants Q667X, Q668X and Y669C adjacent to the V633L variant we report here.^11^ Likewise for RP, the majority of pathogenic variants occur in exon 4^12^ causing both dominant and recessive disease as well as other complex genetic inheritance patterns involving other genes.^13^ The *RP1* variant reported here truncates the protein by just 41 residues and therefore as a result it is only a portion of the very C-terminal disordered domain of the protein that is lost (https://www.uniprot.org/uniprotkb/P56715/entry). How such a small truncation might affect RP1 function remains to be determined. The R702W variant in *NOD2* has been variably associated with CD^9 10^ and in some studies it has been identified as benign (http://mgdd.pasteur.ma/search.php?info=GeneInfo&disease_type=Multifactorial&id_gene=221). Here we hypothesise that these three variants in *WSF1*, *RP1* and *NOD2* lead to the current diagnosis of all three diseases (CC, RP and CD, respectively) in a single member of the pedigree, V-7. Four other family members (IV-2, IV-5, V-3 and V-4) have been identified who harbour the same three sequence variants. All have CC and RP, but we are unaware of their full pathogenic spectrum at this point in time (table 2). It may be that CD has yet to develop or perhaps our single contact who provided the family history for the pedigree presented here is uninformed of their CD diagnosis. It is, however, most likely that CD is a multifactorial disease with a greater weight of environmental exposure required for phenotype development in a polygenetic ‘at risk’ individual within the pedigree.

WS was first described by Wolfram and Wagener.^14^ It is a severe neurodegenerative disease mainly characterised by juvenile-onset diabetes mellitus and optic atrophy along with several other disorders. Wolframin is integral membrane protein within the endoplasmic reticulum (ER),^15^ suggesting a physiological role in membrane trafficking, secretion, processing and/or regulation of ER calcium homeostasis. *WFS1* comprises 8 exons encoding an 890-residue protein called Wolframin. It is a hydrophobic protein with nine transmembrane segments and large hydrophilic regions at both the N-terminal and C-terminal. It can form a tetramer, is glycosylated and locates to the ER.^16^ Disease-causing variants in *WFS1* are known to cause autosomal-recessive WS. Its variable expression in tissues results in a range of clinical complications such as depression, diabetes, hearing and vision loss, bilateral cataract in conjunction with other systemic disorders as well as isolated, dominant bilateral CCs. Wolframin variants are reported to result in ER stress and loss of ER function, disrupting the export of vesicle cargo proteins such as insulin.^17^ To date four autosomal dominant families (including one in this study) and three sporadic cases have been documented with variants in *WFS1* causing isolated cataracts or other WS pathologies^18–21^ (table 3). The majority of recessive *WFS1* inactivating variants cause typical WS, whereas dominant non-inactivating variants are associated with less severe, but more varied, phenotypes. Here, we report a novel missense variant (c.1897G>C; p.V633L) in *WFS1* associated with lamellar CC. V633 is highly conserved in *WFS1* across species, including human, mouse, monkey, rat and chicken (https://www.ncbi.nlm.nih.gov/nuccore/?term=WFS1). Recently, La Morgia and coauthors suggested that *WFS1* variants can affect mitochondrial function and that in turn will affect calcium regulation,^22^ a known cataract risk factor.^23^

**Table 3:** *WFS1* variants associated with CC to date

No.	Exon	HGVSc	HGVSp	Inheritance	Origin	Phenotype	Other defects	Reference
**1**.	Ex4	c.449C>T	p.A150V	AD	China	–	Wolfram-like Syndrome	Fan *et al*^18^
**2**.	Ex6	c.1163T>G	p.L388R	Sporadic	Switzerland	Total	–	Rechsteiner *et al*^19^
**3**.	Ex8	c.1385A>G	p.E462G	AD	Ireland	Nuclear	None	Berry *et al*^4^
**4**.	Ex8	c.1514G>C	p.C505S	AD	China	‘Cauliflower’	Iris coloboma	Li *et al*^20^
**5**.	Ex8	c.2603G>A	p.R868H	Sporadic	China	Subcapsular	Wolfram-like Syndrome	Fan *et al*^18^
**6**.	Ex8	c.2722G>A	p.E908K	Sporadic		–	Wolfram-like Syndrome	Prochazkova *et al*^21^
**7**.	**Ex8**	**c.1897G>C**	**p.V633L**	**AD**	**UK**	**Lamellar**	–	**Present study**

Bold values indicate the study in question.

AD, Autosomal Dominant; CC, congenital cataract; HGVSc, Human Genome Variation Society (codon); HGVSp, Human Genome Variation Society (protein).

Furthermore, in the above pedigree, the *RP1* variant p.L2115* was found in exon 4, along with the *WFS1* and *NOD2* variants, causing late onset RP in all family members who harboured this variant. *RP1* comprises four exons and encodes a 2156-residue protein. RP is a clinically and genetically heterogeneous group of progressive inherited retinal disorders causing retinal degeneration and affecting 1 in 3000 people.^24^ The clinical variation reflects differences in the age of onset, progression, retinal appearance and visual outcome.^12^ Pathogenic variants in *RP1* often lead to a truncated protein, but a splice site variant in a long non-coding RNA was linked to Volkmann cataract, where opacities are formed around the lens sutures.^25^ This indicates the potential importance of *RP1* to the lens as well as the retina. Indeed, *RP1* is expressed in the postnatal mouse lens epithelium (https://research.bioinformatics.udel.edu/iSyTE/ppi/) and should be considered as a CC causing gene.

Through correspondence with a single family member, we became aware that one individual (V-7) had presented with severe CD in their early 20s. Later, we found four other family members carrying the *NOD2* pathogenic variant, but we are currently unaware of any clinical diagnosis of CD as mentioned earlier. CD is a chronic inflammatory disease of the gastrointestinal tract affecting 26–200 per 100 000 in European populations. The exact cause of CD is unknown, though it is likely to involve a disrupted immunological response to gut microbiota in genetically susceptible individuals.^26^ Genetic studies have begun to shed light on CD. Many of the major genes related to CD, including *NOD2, ATG16L1, IL23R* and *IRGM*, are involved in immune system function^27^ and are induced by the presence of bacteria in the lining of the digestive tract.^28^ Recently, it has also been shown that store-operated calcium entry regulates immune cell function in inflammatory bowel disease.^29^ This is a potential link to CD contributing to exacerbation of the symptoms/time of onset as seen in the case of the affected individual (V-7; Figure 1).

With the emergence of NGS technology, multiple disease genes are being found in the same family. Digenic inheritance was first reported in 1994 for human RP^30^ followed by Bardet-Biedl syndrome.^31^ Recently, digenic inheritance of disease-causing variants in *EPHA2* and *SLC26A4* have also been reported in Pendred syndrome.^32^ Recently, Kinoshita and coauthors^33^ described trigenic *ADH5*/*ALDH2*/*ADGRV1* mutations in myelodysplasia with Ushers syndrome.

In summary, herein we report the putative trigenic inheritance of *WFS1*, *RP1* and *NOD2* variants causing lamellar CC, autosomal dominant RP and CD as multimorbidity phenotypes in a single British pedigree. No one in the pedigree with CC, who was available for genotyping, presented only with the WSF1 variant. All with CC and the WSF1 variant presented with either *RP1* (III-2, IV-7, V-2, V-5 and V-6) or *NOD2* (V-3 or both (IV-2, IV-, V-4; V-7) accompanying WSF1 (figure 1). We speculate that there might be a link to CD as a result of disrupted calcium regulation. The literature suggests that ocular phenotypes accompany CD^34^ identifying multimorbidities as increasingly important for the development of personalised medicines and treatment strategies. However, we cannot at this point in time provide functional studies to support the genetic associations reported in this study.

## Data Availability

Data are available upon reasonable request.
